# FSCPX, a Chemical Widely Used as an Irreversible A_1_ Adenosine Receptor Antagonist, Modifies the Effect of NBTI, a Nucleoside Transport Inhibitor, by Reducing the Interstitial Adenosine Level in the Guinea Pig Atrium

**DOI:** 10.3390/molecules23092186

**Published:** 2018-08-30

**Authors:** Tamas Erdei, Adrienn Monika Szabo, Nora Lampe, Katalin Szabo, Rita Kiss, Judit Zsuga, Csaba Papp, Akos Pinter, Andras Jozsef Szentmiklosi, Zoltan Szilvassy, Bela Juhasz, Rudolf Gesztelyi

**Affiliations:** 1 Department of Pharmacology and Pharmacotherapy, Faculty of Medicine, University of Debrecen, Nagyerdei krt. 98, H-4032 Debrecen, Hungary; erdei.tamas@pharm.unideb.hu (T.E.); lampenori@gmail.com (N.L.); szabo.katalin@pharm.unideb.hu (K.S.); kiss.rita@med.unideb.hu (R.K.); ajszm948@gmail.com (A.J.S.); szilvassy.zoltan@med.unideb.hu (Z.S.); juhasz.bela@med.unideb.hu (B.J.); 2 Department of Internal Medicine (Building C), Faculty of Medicine, University of Debrecen, Nagyerdei krt. 98, H-4032 Debrecen, Hungary; szabo.adrienn23@gmail.com; 3 Department of Health Systems Management and Quality Management for Health Care, Faculty of Public Health, University of Debrecen, Nagyerdei krt. 98, H-4032 Debrecen, Hungary; zsuga.judit@med.unideb.hu (J.Z.); dr.papp.csaba@gmail.com (C.P.); 4 Institute of Mathematics, Faculty of Science and Technology, University of Debrecen, Egyetem ter 1, H-4032 Debrecen, Hungary; apinter@science.unideb.hu

**Keywords:** adenosine, CPA, FSCPX, NBTI, A_1_ adenosine receptor, receptor reserve, heart, atrium, receptorial responsiveness method, RRM

## Abstract

Based on *in silico* results, recently we have assumed that FSCPX, an irreversible A_1_ adenosine receptor antagonist, inhibits the action of NBTI that is apparent on *E*/*c* curves of adenosine receptor agonists. As a mechanism for this unexpected effect, we hypothesized that FSCPX might modify the equilibrative and NBTI-sensitive nucleoside transporter (ENT1) in a way that allows ENT1 to transport adenosine but impedes NBTI to inhibit this transport. This assumption implies that our method developed to estimate receptor reserve for agonists with short half-life such as adenosine, in its original form, overestimates the receptor reserve. In this study, therefore, our goals were to experimentally test our assumption on this effect of FSCPX, to improve our receptor reserve-estimating method and then to compare the original and improved forms of this method. Thus, we improved our method and assessed the receptor reserve for the direct negative inotropic effect of adenosine with both forms of this method in guinea pig atria. We have found that FSCPX inhibits the effects of NBTI that are mediated by increasing the interstitial concentration of adenosine of endogenous (but not exogenous) origin. As a mechanism for this action of FSCPX, inhibition of enzymes participating in the interstitial adenosine production can be hypothesized, while modification of ENT1 can be excluded. Furthermore, we have shown that, in comparison with the improved form, the original version of our method overestimates receptor reserve but only to a small extent. Nevertheless, use of the improved form is recommended in the future.

## 1. Introduction

Irreversible antagonists play a pivotal role in the investigation of receptors, being useful for differentiating receptor types and subtypes and being essential in the assessment of receptor reserve. Receptor reserve is an integrative measure of the response-inducing ability of the interaction between an agonist and receptor system consisting of a receptor and postreceptorial signalling [1,2]. This term is coined to elucidate the phenomenon, that is, in some cases, the stimulation of a submaximal fraction of receptors can apparently elicit the maximal effect. Accordingly, detection and quantification of receptor reserve is based on the comparison of *E*/*c* curves generated in the absence and presence of an irreversible receptor antagonist. As receptor reserve depends on the tissue, agonist and effect, receptor reserve values should be determined for each tissue, agonist and effect being of practical importance [1,2].

The A_1_ adenosine receptor (A_1_ receptor) is a member of the ancient and ubiquitous adenosine receptor family that exerts complex regulatory functions in almost all tissues [3,4,5]. In the heart, the A_1_ receptor mediates strong negative tropic effects including negative inotropy on the ventricle and, even more, on the atrium [6], as a component of the A_1_ adenosinergic cardioprotection against ischemic/hypoxic stress [7,8,9]. Accordingly, in earlier studies, we aimed to assess the guinea pig atrial A_1_ receptor reserve for the direct negative inotropic effect of several A_1_ adenosine receptor agonists (the term direct means that experiments were conducted on atria lacking prior stimulation of contractility). For this purpose, concentration-response (*E*/*c*) curves were constructed in the absence and presence of 8-cyclopentyl-*N*^3^-[3-(4-(fluorosulfonyl)benzoyloxy)propyl]-*N*^1^-propylxanthine (FSCPX). After its first appearance in a report from 1994 [10], FSCPX was tested and then utilized as a selective and irreversible A_1_ receptor antagonist in numerous investigations [11,12,13,14,15,16,17,18,19].

In the case of stable, synthetic full agonists (such as *N*^6^-cyclopentyladenosine: CPA), the A_1_ receptor reserve could be successfully determined with the operational model of agonism and Furchgott’s method, two procedures providing a quantitative result. The A_1_ receptor reserve proved to be extremely large for the direct negative inotropic effect [17]. This finding could be well illustrated by the fact that FSCPX was unable to significantly decrease the maximal effects of these agonists, although, based on results of Furchgott’s method, about 80–90% of the A_1_ receptors were inactivated by FSCPX. Instead, FSCPX only shifted the *E*/*c* curves of the stable agonists to the right, as if it were a competitive antagonist [17]. However, our attempt failed to assess the A_1_ receptor reserve for the direct negative inotropic effect of adenosine, the physiological agonist, when naïve and FSCPX-pre-treated adenosine *E*/*c* curves were evaluated with the operational model and Furchgott’s method. This failure was probably due to the too short half-life of adenosine in our ex vivo experimental system that impeded adenosine *E*/*c* curves to get saturated [17].

To address this challenge, an alternative receptor reserve-estimating method had to be developed for adenosine [18]. The essence of this method is the use of *S*-(2-hydroxy-5-nitrobenzyl)-6-thioinosine (NBTI), a selective inhibitor of the equilibrative and NBTI-sensitive nucleoside transporter (ENT1; SLC29A1) [20]. As the physiological adenosine transport is directed into the cells (e.g., cardiomyocytes) [21,22], NBTI prevents adenosine, administered for the *E*/*c* curve, from the intracellular degradation and reutilization, allowing enough time for the exogenous adenosine to exert its effect. However, NBTI also prevents endogenous adenosine from the intracellular elimination that leads to a rise of the interstitial adenosine concentration. Importantly, this latter phenomenon occurs already before the generation of the adenosine *E*/*c* curve (and it exists throughout the experiment, till NBTI is present). As the surplus endogenous adenosine accumulated by NBTI consumes in part the response capacity of A_1_ receptors (and their postreceptorial signalling) before constructing the adenosine *E*/*c* curve, the response to adenosine (detected by the given adenosine *E*/*c* curve) will show an apparent diminution. Thus, effect values of adenosine *E*/*c* curves generated in the presence of NBTI should be corrected for the distortion caused by the increased endogenous adenosine level in the interstitium. For this correction, we apply a procedure based on the receptorial responsiveness method (RRM) [23,24]. Finally, the corrected FSCPX-naïve and FSCPX-pre-treated adenosine *E*/*c* curves (generated in the presence of NBTI) are compared. Information on the receptor reserve in question can be obtained from the distance of the final (saturated) parts of the corrected curves: small distance means great receptor reserve, while large distance indicates a small one. Although our receptor reserve-estimating method provides only qualitative results, the A_1_ receptor reserve for the direct negative inotropic effect of adenosine could be assessed and has been found similarly large as that for the synthetic agonists, in both eu- [18] and hyperthyroid states [19].

However, during the process of determining receptor reserve with our qualitative method, we encountered an astonishing phenomenon that, in the presence of NBTI, FSCPX pre-treatment apparently increased the response to adenosine (when the FSCPX-NBTI co-treated adenosine *E*/*c* curve was compared to the solely NBTI-treated one) [18,19]. Starting from this pharmacological paradox, a recent *in silico* study from our laboratory has raised the possibility that FSCPX pre-treatment may modify the effect of NBTI through a mechanism other than blocking the A_1_ receptor [25]. To the best of our knowledge, no other paper has reported any effect of FSCPX other than permanent A_1_ receptor inactivation (even as a hypothesis).

Our assumption about an interference between effects of FSCPX and NBTI implies that our receptor reserve-estimating method should be revisited. In the present study, therefore, our primary goal was to justify or refute our assumption about this interference. Furthermore, our goal was to improve our receptor reserve-estimating method to eliminate the distorting effect resulted from this unexpected interference. Finally, we aimed to compare results obtained from the original and improved forms of our method. For these purposes, we have included a new protocol to the old ones used in previous studies (i.e., [18,19]) (see: Table 1). This amendment has offered new information on the mechanism of action of FSCPX, furthermore it has made our receptor reserve-estimating method free of the disturbing effect stemming from the interference between actions of FSCPX and NBTI.

## 2. Results

### 2.1. Naïve Adenosine and CPA E/c Curves (Serving as a Control)

**Figure 1:**
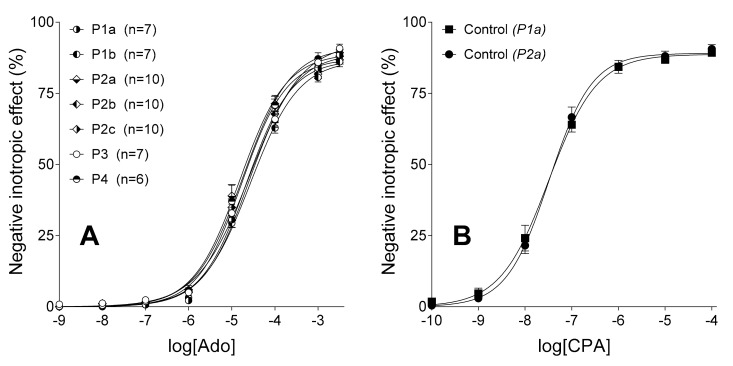
The direct negative inotropic effect of adenosine (panel **A**) and CPA (panel **B**) in the absence of any chemicals modifying the adenosinergic homeostasis in the isolated guinea pig atrium. In the (panel **A**), the first concentration-response (*E*/*c*) curve, generated with adenosine, is shown in all the seven groups (P1a, P1b, P2a, P2b, P2c, P3 and P4). In the (panel **B**), the second *E*/*c* curve, constructed with CPA, is presented in groups P1a and P2a. The term “Control” refers to the role of these curves and also indicates their treatment status (naïve). The *x*-axis denotes the common logarithm of the molar concentration of the agonists (in the bathing medium) and the *y*-axis indicates the effect (as a percentage decrease in the initial contractile force). The symbols show the responses to the agonists averaged within the groups (±SEM) and the curves illustrate the fitted Hill equation (Equation (1)). Ado: adenosine; CPA: *N*^6^-cyclopentyladenosine.

Adenosine concentration-dependently decreased the contractile force of all atria (Figure 1A). Empirical parameters of the first adenosine *E*/*c* curves, provided by fitting of the Hill equation (Equation (1)), did not show any significant differences among the experimental groups (data not shown). This observation indicates the homogeneity of atria used for this investigation.

CPA also reduced the atrial contractile force in a concentration-dependent manner (Figure 1B). Empirical parameters of CPA *E*/*c* curves of groups P1a and P2a did not differ significantly from each other (Table 2), indicating that differences between their protocols (especially in duration, see: Table 1) did not affect the response to CPA.

### 2.2. CPA E/c Curves in Groups P1 and P2

**Figure 2:**
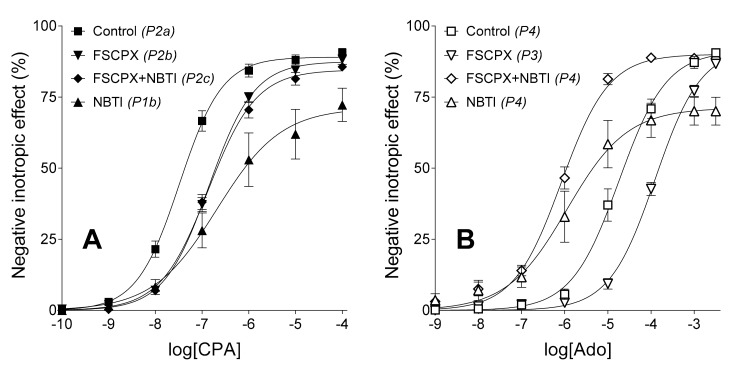
The direct negative inotropic effect of CPA (panel **A**) and adenosine (panel **B**) without and with a previous FSCPX pre-treatment, combined with the absence and presence of NBTI. The applied (pre)treatments and experimental groups (*latter ones in italics*) are indicated. For simplicity, the P1a (Control) curve and P3 Control curve (not differing significantly from the P2a (Control) curve and P4 Control curve, respectively) are omitted. The *x*-axis denotes the common logarithm of the molar concentration of the agonists (in the bathing medium) and the *y*-axis indicates the effect (as a percentage decrease in the initial contractile force). The symbols show the responses to the agonists averaged within the groups (±SEM) and the curves denote the fitted Hill equation (Equation (1)). Ado: Adenosine; CPA: *N*^6^-cyclopentyladenosine; NBTI: *S*-(2-hydroxy-5-nitrobenzyl)-6-thioinosine; FSCPX: 8-cyclopentyl-*N*^3^-[3-(4-(fluorosulfonyl)benzoyloxy)propyl]-*N*^1^-propylxanthine.

Consistent with our earlier observations [18,19,26,27], NBTI treatment markedly pushed down the CPA *E*/*c* curve (decreasing *E*_max_) and shifted it to the right (increasing log*EC*_50_), as compared to its control CPA curve. Also in agreement with our previous findings [17,18,19], FSCPX pre-treatment caused a moderate rightward shift of the CPA *E*/*c* curve (only increasing log*EC*_50_ in comparison with its control CPA curve). However, combination of FSCPX and NBTI made the CPA *E*/*c* curve almost the same as the solely FSCPX-pre-treated one, aside from a slight, non-significant reduction of *E*_max_ and Hill coefficient. Thus, FSCPX pre-treatment almost entirely abolished all effects of NBTI on the response to CPA (Figure 2A, Table 2).

**Figure 3:**
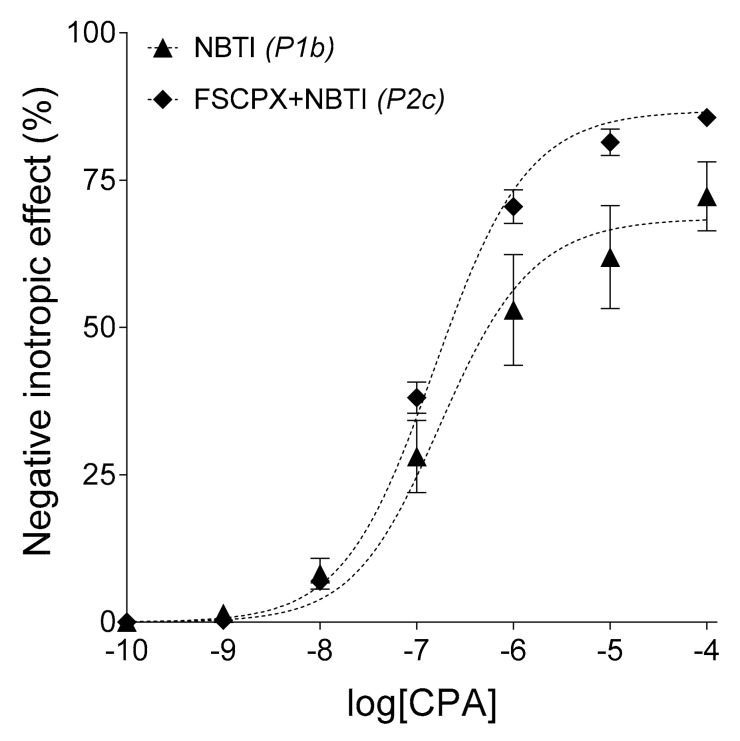
The direct negative inotropic effect of CPA in the presence of NBTI, without and with a previous FSCPX pre-treatment. The applied (pre)treatments and experimental groups (*latter ones in italics*) are indicated. The *x*-axis denotes the common logarithm of the molar CPA concentration (in the bathing medium) and the *y*-axis indicates the effect (as a percentage decrease in the initial contractile force). The symbols show the responses to CPA averaged within the groups (±SEM) and the dotted curves illustrate the fitted model of RRM (Equation (2)). CPA: *N*^6^-cyclopentyladenosine; NBTI: *S*-(2-hydroxy-5-nitrobenzyl)-6-thioinosine; FSCPX: 8-cyclopentyl-*N*^3^-[3-(4-(fluorosulfonyl)benzoyloxy)propyl]-*N*^1^-propylxanthine.

The surplus interstitial adenosine accumulated by NBTI was found to be equieffective with 100.2 nM and 6.73 nM CPA in the P1b and P2c groups, respectively. These outcomes came from best-fit values provided by RRM as log(*c_x_*) values being −6.99 and −8.17 (with a 95% confidence interval from −7.18 to −6.84 and from −∞ to −7.72), respectively (Figure 3). Thus, FSCPX pre-treatment reduced the concentration of the extra interstitial adenosine produced by NBTI to less than a tenth in the isolated guinea pig atrium.

### 2.3. Adenosine E/c Curves in Groups P3 and P4

As expected (based on References [18,19]), NBTI caused a significant depression and substantial sinistral displacement in the adenosine *E*/*c* curve, as compared to its control adenosine curve (although the decrease of *E*_max_ was greater than previously observed). Also in line with expectations (based on [17,18,19]), FSCPX pre-treatment shifted the adenosine *E*/*c* curve to the right as compared to its control adenosine curve, approximately to an extent as it did with the CPA *E*/*c* curve. At the same time, the FSCPX-NBTI co-treatment produced an adenosine *E*/*c* curve, whose *E*_max_ practically equalled *E*_max_ of the control as well as solely FSCPX-pre-treated adenosine *E*/*c* curves, while log*EC*_50_ of which was similar to log*EC*_50_ of the exclusively NBTI-treated adenosine *E*/*c* curve. So, FSCPX pre-treatment appeared to inhibit some but not all the effects of NBTI on the response to adenosine (Figure 2B, Table 2).

### 2.4. Interference of FSCPX Pre-Treatment with NBTI

Previously, we observed that, in the presence of NBTI, FSCPX pre-treatment slightly (but clearly) enhanced the response to adenosine [18,19]. In the present investigation, the extent of this interference was unexpectedly large. FSCPX pre-treatment significantly augmented the response to both CPA and adenosine not only at high concentrations but also at medium ones, when contrasted the FSCPX-NBTI co-treated *E*/*c* curves with the solely NBTI-treated ones (Figure 2A,B). Nevertheless, FSCPX pre-treatment did not significantly affect the extensive leftward shift of the adenosine *E*/*c* curve caused by NBTI. As a result, the FSCPX-NBTI co-treated CPA *E*/*c* curve and the FSCPX-pre-treated one almost coincide, whereas the FSCPX-NBTI co-treated adenosine *E*/*c* curve and the FSCPX-pre-treated one are furthest apart (Figure 2A,B, Table 2).

Another way to show this interference, if we contrast the differences between the NBTI-treated *E*/*c* curves and the corresponding control *E*/*c* curves with the differences between the FSCPX-NBTI co-treated *E*/*c* curves and the corresponding FSCPX-pre-treated *E*/*c* curves. In this manner, atria possessing similar operable A_1_ receptor population can be compared. Regarding the CPA *E*/*c* curves, NBTI alone decreased *E*_max_ by about 16.7% and produced a 10-fold shift of the *E*/*c* curve to the right. In contrast, after FSCPX pre-treatment, NBTI decreased *E*_max_ only by 2.9% and did not shift the *E*/*c* curve (Table 2). In the case of the adenosine *E*/*c* curves, NBTI alone reduced *E*_max_ by 19.8% and caused a 15-fold shift of the *E*/*c* curve to the left. After FSCPX pre-treatment, however, NBTI decreased *E*_max_ by 3.6% (similarly to results with CPA), while produced a huge, about 158-fold shift to the left (Table 2).

### 2.5. Corrected Effects of Adenosine E/c Curves Generated in the Presence of NBTI

**Figure 4:**
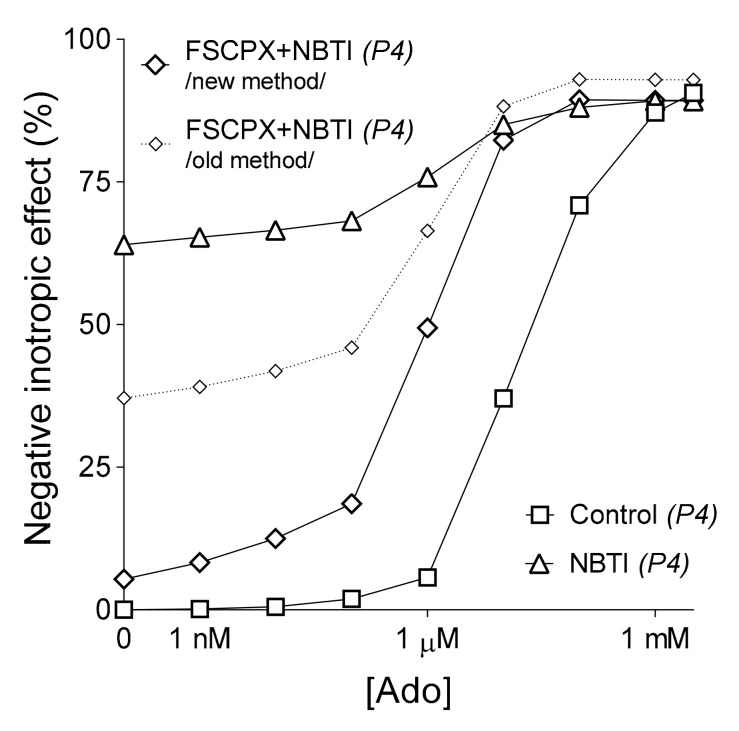
The corrected effect values of the adenosine concentration-response (*E*/*c*) curves in the P4 group constructed in the presence of NBTI (without and with a previous FSCPX pre-treatment), furthermore the original effect values of the averaged, control adenosine *E*/*c* curve in the P4 group (considered to be inherently correct). The applied (pre)treatments and the experimental group (*latter one in italics*) are indicated. The *x*-axis shows the adenosine concentrations (in the bathing medium) on a logarithmic scale and the *y*-axis indicates the effect (as a percentage decrease of the initial contractile force). The symbols represent the responses to adenosine averaged within the groups. Nota bene: While effect values of *E*/*c* curves generated in the presence of NBTI (no matter with or without FSCPX) were evoked by adenosine of both (exogenous and endogenous) origins, they could be plotted only *versus* the exogenous adenosine levels in the bathing medium of atria. New method: our receptor reserve-estimating method used in its improved form; old method: our receptor reserve-estimating method applied in its original form [18]; Ado: Adenosine; NBTI: *S*-(2-hydroxy-5-nitrobenzyl)-6-thioinosine; FSCPX: 8-cyclopentyl-*N*^3^-[3-(4-(fluorosulfonyl)benzoyloxy)propyl]-*N*^1^-propylxanthine.

The averaged, solely NBTI-treated adenosine *E*/*c* curve of the P4 group containing effect values corrected for the bias caused by the extra interstitial adenosine accumulated by the NBTI, shortly the corrected NBTI-treated adenosine *E*/*c* curve, substantially exceeded its uncorrected (conventionally evaluated) counterpart. The maximum of the corrected NBTI-treated adenosine *E*/*c* curve reached that of its control (the naïve adenosine *E*/*c* curve of the P4 group). The starting point of the corrected NBTI-treated adenosine *E*/*c* curve was the effect evoked by the surplus interstitial adenosine produced by NBTI in the absence of FSCPX (*E*_x_ belonging to *c_x_* = 100.2 nM CPA) (Figure 4).

The averaged, FSCPX-NBTI co-treated adenosine *E*/*c* curve of the P4 group was corrected two ways: With an improved method based on findings of our recent *in silico* study [25] and with our original method reported earlier [18].

When computed using the improved method, the corrected FSCPX-NBTI co-treated adenosine *E*/*c* curve started deep beneath the corrected NBTI-treated adenosine *E*/*c* curve but reached practically the same maximum as its control curve and the corrected NBTI-treated adenosine *E*/*c* curve did. In agreement with our previous finding [18,19], this indicates the existence of a great receptor reserve for the direct negative inotropic effect of adenosine in the guinea pig atrium. The starting point of the corrected FSCPX-NBTI co-treated adenosine *E*/*c* curve was the effect elicited by the surplus interstitial adenosine produced by the NBTI in the presence of FSCPX (*E_x_* belonging to *c_x_* = 6.73 nM CPA) (Figure 4).

The FSCPX-NBTI co-treated adenosine *E*/*c* curve that was corrected with the original method ran noticeably above its counterpart corrected with the improved method at low and medium (exogenous) adenosine concentrations, while it exceeded its improved counterpart to a small extent at high (exogenous) adenosine concentrations (Figure 4). This finding shows that results obtained from the original method overestimate the receptor reserve in question but only in a small compass.

It should be noted that, as exact values of the endogenous (and thereby the total) interstitial adenosine concentrations at the A_1_ receptors remained unknown throughout the investigation, the corrected effect values could only be plotted versus the concentrations of exogenous adenosine in the bathing medium (that could be easily calculated). Nevertheless, within the P4 group, the inherently correct (i.e., NBTI-free) effect values and the corrected ones (of course belonging to the same exogenous adenosine concentration) can be meaningfully compared to one another (Figure 4).

## 3. Discussion

The main experimental finding of the present study is that a pre-treatment with FSCPX, a chemical widely considered to be a selective and irreversible A_1_ receptor antagonist, selectively influences the different effect components of NBTI, a selective nucleoside transport inhibitor, that are apparent on *E*/*c* curves of adenosine and CPA, two A_1_ receptor agonists, in the isolated guinea pig atrium. While FSCPX pre-treatment considerably counteracts the depressive (*E*_max_-decreasing) effect of NBTI on *E*/*c* curves of both adenosine and CPA, it does not significantly affect the extensive action of NBTI on *EC*_50_ of the adenosine *E*/*c* curve. Thus, our recent proposal, that is, FSCPX blunts the effect of NBTI [25], should be refined in light of the new results. It seems that FSCPX pre-treatment inhibits the effects of NBTI that are mediated by elevating the interstitial level of endogenous but not exogenous adenosine. Consequently, the target of FSCPX (other than the A_1_ receptor) cannot be the ENT1 transporter or any other molecules that participate in the mediation of the effect of NBTI on the level of exogenous adenosine. Conversely, the target in question may be a (or some) molecule(s) that is (are) associated exclusively with the effect of NBTI exerted on the level of endogenous adenosine, for example, enzymes contributing to the interstitial adenosine formation.

In a recent investigation dealing with the *in silico* modelling of our receptor reserve-estimating method [25], we have found that *E*/*c* curves resulted from studies using this method [18,19] could not be simulated without supposing an interference between effects of FSCPX and NBTI. Based on this finding, we hypothesized that FSCPX might modify ENT1 in a way that it allows ENT1 to transport adenosine but impedes NBTI to inhibit this transport [25]. In the present study, in order to explore the interference between effects of FSCPX and NBTI, we have amended the protocols of our receptor reserve-estimating method to enable detecting the influence of FSCPX and NBTI co-treatment on the CPA *E*/*c* curve.

The effects of FSCPX and NBTI on the *E*/*c* curves of adenosine receptor agonists can be divided into three components: Effects mediated by influencing the interstitial level of endogenous adenosine (that were earlier attributed exclusively to NBTI [26,27]), effects mediated *via* affecting the interstitial level of exogenous adenosine (also ascribed to NBTI [28]) and effects resulted from the permanent inactivation of a fraction of the A_1_ receptor population (attributed to FSCPX [11]).

In terms of the shape of our *E*/*c* curves, differentiation of effects of “endogenous” and “exogenous” adenosine originates in the features of our experimental model. The “effects mediated by influencing the interstitial level of endogenous adenosine” refers to consequences of a rise in the interstitial adenosine concentration developed ***before*** the construction of the *E*/*c* curve of an adenosine receptor agonist. This occurs when NBTI, administered to the atria before the generation of the *E*/*c* curve, reduces the elimination of adenosine produced interstitially in the course of the tissue adenosine turnover. In contrast, the “effects mediated via affecting the interstitial level of exogenous adenosine” means outcomes of an attenuated decrease (so a relative increase) in the interstitial adenosine concentration ***during*** the construction of an adenosine *E*/*c* curve. This is the case when NBTI decreases the elimination of adenosine administered as *E*/*c* curve doses. The point is that, if receptors (with their signalling) respond to a stimulus before the generation of an *E*/*c* curve, this response will be reflected in the subsequent *E*/*c* curve as a deficit in the responsiveness of the receptors (and their signalling). This is the motif of RRM [23,24] and that is why RRM can be used to correct *E*/*c* curves distorted by a response evoked before the construction of the *E*/*c* curve [18]. As a result, increase in interstitial concentrations of endogenous and exogenous adenosine (upon our experimental conditions) exert the opposite effect on the main parameters (*E*_max_, *EC*_50_) of an adenosine *E*/*c* curve (Table 3).

In the case of an NBTI-treated CPA *E*/*c* curve, the situation is relatively simple: The surplus endogenous adenosine produced by NBTI will consumes the response capacity of the cell-surface A_1_ receptors (and their postreceptorial pathways) before the construction of the *E*/*c* curve, therefore NBTI decreases *E*_max_ and increases *EC*_50_ of the CPA *E*/*c* curve [26]. In the case of an NBTI-treated adenosine *E*/*c* curve, the situation is more complex: Consequences of the elevated endogenous adenosine level will combine with effects stemming from the preservation of adenosine administered for the *E*/*c* curve, which latter effects tend to increase *E*_max_ and to decrease *EC*_50_ of the adenosine *E*/*c* curve. As a resultant, NBTI reduces both *E*_max_ and *EC*_50_ of the adenosine *E*/*c* curve [28] (Table 3).

Before the emergence of our recent results [25], action of FSCPX appeared the simplest: similar increase in *EC*_50_ of the CPA and adenosine *E*/*c* curves, consistent with the irreversible A_1_ receptor antagonist property of FSCPX and the large A_1_ receptor reserve for the direct negative inotropy in the guinea pig atrium [17,18,19]. In the present study, however, when we applied an FSCPX pre-treatment before the construction of the CPA *E*/*c* curve in the presence of NBTI, the resulting effect was an almost complete cancelling of NBTI’s effects with the preservation of the action of FSCPX (Figure 2A, Table 2). This curve constellation cannot be explained with the irreversible A_1_ receptor antagonist property of FSCPX. If FSCPX were a simple irreversible antagonist, it would have only shifted the NBTI-treated CPA *E*/*c* curve to the right (increasing its *EC*_50_). In turn, when an FSCPX-NBTI co-treatment was applied in the case of an adenosine *E*/*c* curve, the *E*_max_-decreasing effect of NBTI was only cancelled, while the *EC*_50_-decreasing effect of NBTI remained practically intact (Figure 2B, Table 2). This curve constellation further contradicts the prevailing concept being FSCPX only an irreversible A_1_ receptor antagonist, moreover it also shows that the additional effect of FSCPX cannot be an equal inhibition of all effects of NBTI.

Based on *in silico* results, recently we hypothesized that FSCPX might impede the inhibitory action of NBTI on ENT1 [25]. It should be reminded that all effects of NBTI stem from the inhibition of ENT1 [20], by which NBTI reduces the physiologically inward transmembranous adenosine transport and thus increases the interstitial concentration of adenosine of any origin [21,22]. The above-mentioned hypothesis would agree with the present results obtained from CPA *E*/*c* curves (Figure 2A, Table 2) but it contrasts with the current results yielded from adenosine *E*/*c* curves (Figure 2B, Table 2). Such a great increase in *E*_max_ together with the preservation of *EC*_50_, seen in the case of the FSCPX-NBTI co-treated adenosine *E*/*c* curve as compared to the only NBTI-treated one, can hardly be imagined if we suppose the inhibition of all effects of NBTI (and inhibition of ENT1 would decrease all NBTI-evoked effects). Interestingly, earlier studies [18,19] yielded results, for which this previous hypothesis appeared to work. However, depression of the solely NBTI-treated adenosine *E*/*c* curve in those studies was considerably smaller than that in the present investigation, although relative positions of the control, FSCPX-pre-treated, NBTI-treated and FSCPX-NBTI co-treated adenosine *E*/*c* curves were the same in all above-mentioned studies.

Taking all together, the target of FSCPX, to exert its effect other than A_1_ receptor inactivation, should be a molecule that is related to the effect of NBTI that is mediated by increasing the interstitial level of endogenous adenosine. Enzymes contributing (exclusively or at least predominantly) to the interstitial adenosine formation are possible candidates. It can be speculated that FSCPX, which ruins the binding site for adenosine in the A_1_ receptor, can do the same with the binding site of one (or some) enzyme(s) participating in the interstitial adenosine production. If it were so, FSCPX pre-treatment would decrease the interstitial adenosine level. As the resting interstitial adenosine concentration elicits undetectably small inotropic effect in the guinea pig atrium [29], the supposed diminution of it by FSCPX would be also undetectable. However, under conditions with reduced interstitial adenosine producing capacity resulted from an FSCPX pre-treatment, NBTI could elevate the interstitial level of endogenous adenosine to a smaller extent than otherwise. This reduced increase in the interstitial adenosine level (before generating an *E*/*c* curve) would cause easily detectable alterations in *E*/*c* curves of both adenosine and CPA: Tendency to a smaller decrease in *E*_max_ and to a smaller increase in *EC*_50_ (Table 3). It is also important that, supposing the above-mentioned additional mechanism of action for FSCPX, the protective effect of NBTI on the exogenous adenosine would freely prevail. Regarding the shape of our CPA and adenosine *E*/*c* curves (Figure 2A,B, Table 2), this is exactly the case in the present investigation. The only concern might be the fact that, after FSCPX pre-treatment, NBTI did not cause a significantly greater decrease in *EC*_50_ of the adenosine *E*/*c* curve as compared to the control state than NBTI alone. However, this phenomenon can be ascribed to the irreversible antagonist property of FSCPX. When comparing the FSCPX-NBTI co-treated adenosine *E*/*c* curve to the solely FSCPX-pre-treated one, the sinistral displacement (decrease in *EC*_50_) is much larger than it is if comparing the NBTI-treated adenosine *E*/*c* curve to the control one (Figure 2A,B, Table 2). Thus, we have improved our hypothesis that is (from the appearance of this writing) as follows: FSCPX pre-treatment modifies the action of NBTI on *E*/*c* curves of adenosine receptor agonists via limiting the interstitial adenosine producing capacity, perhaps by inhibiting one (or some) interstitial adenosine forming enzyme(s) (Table 3). (Theoretically, an alternative mechanism for FSCPX to decrease the interstitial adenosine level could be the enhancement of elimination of interstitial adenosine. However, this mechanism is unlikely because it would lower the concentration of both exogenous and endogenous adenosine, a possibility that is not supported by the present results)

A phenomenon that is also worthwhile to address is the slight increase of the response to CPA at low concentrations in the case of the FSCPX-NBTI co-treated CPA *E*/*c* curve, as compared to its adequate counterpart, the FSCPX-pre-treated CPA *E*/*c* curve (Figure 2A). This may be explained by supposing that CPA, in agreement with our previous observation [23], undergoes a minor elimination in the atrial tissue. As in the case of the NBTI-treated and FSCPX-NBTI co-treated adenosine *E*/*c* curves in comparison with the control and FSCPX-pre-treated ones, respectively (Figure 2B), the increased response to the given adenosine receptor agonist shows the protective effect of NBTI that manifests predominantly at low concentrations (Figure 2A,B). Thus, for further studies in this topic, use of *N*^6^-cyclohexyladenosine (CHA), a selective A_1_ receptor full agonist showing higher resistance against adenosine-handling enzymes [30], should be considered.

Further observation of the present study is that the scatter of *E*/*c* curve data is the biggest for the solely NBTI-treated curves. Interestingly, this big scatter caused by NBTI has been largely prevented by the FSCPX-pre-treatment (Figure 2A,B, Figure 3). It may be speculated that the cause of the exceptionally big scatter in the presence of NBTI is due to the considerable individual variability of enzyme activities contributing to the interstitial adenosine production that, if inhibited by FSCPX, makes the response to adenosine receptor agonists more homogenous.

A godsend for this investigation was that NBTI exerted very strong *E*_max_-decreasing (for both CPA and adenosine *E*/*c* curves: Figure 2A,B) and *EC*_50_-increasing (for the CPA *E*/*c* curve: Figure 2A) effects during the experiments presented herein, in comparison with earlier studies [18,19,26,27]. In our interpretation, these effects of NBTI on the *E*/*c* curves of adenosine receptor agonists can be ascribed to the action of NBTI on the interstitial level of endogenous adenosine (Table 3). Thus, it is reasonable to assume that, in our present experiments, atria possessed exceptionally high interstitial adenosine forming capacity, allowing both NBTI and FSCPX to exert especially strong effects on the interstitial level of endogenous adenosine. Consistently, we have found the highest *c_x_* value ever in our praxis that characterizes well the efficiency of NBTI in the present study (Table 4). For the reason mentioned in the previous paragraph, the efficiency of FSCPX (to elicit its additional effect) could only manifest in its interaction with NBTI: The FSCPX pre-treatment produced a considerably large diminution in *c_x_* (from 100.2 nM to 6.73 nM). These strong effects observed concerning FSCPX and NBTI enabled us to improve our previous assumption on the mechanism of action of FSCPX other than A_1_ receptor inactivation.

The *c_x_* values provided the opportunity to create hypothetical adenosine *E*/*c* curves free of the disturbing effect of the interstitial accumulation of endogenous adenosine in response to NBTI (Figure 4). These hypothetical adenosine *E*/*c* curves allow us to compare our improved receptor reserve-estimating method with the original one. The FSCPX-NBTI co-treated adenosine *E*/*c* curve, corrected with the original method, ran unequivocally above its counterpart corrected with the improved method (Figure 4). This phenomenon corroborates the assumption made previously [25] that the original method, used for two earlier studies [18,19], overestimates and thereby overcorrects the distortion caused by NBTI, if previous FSCPX pre-treatment has occurred. Since this inaccuracy affects responses belonging to the high adenosine concentrations only to a small extent and since these responses form the basis to estimate receptor reserve (via comparing them with the corresponding response values of the corrected NBTI-treated adenosine *E*/*c* curve [18,19]), the original method over measures the receptor reserve in question only to a slight extent (Figure 4). Of course, application of the improved method is recommended in the future.

## 4. Materials and Methods

### 4.1. Materials

The following chemicals were used: Adenosine, *N*^6^-cyclopentyladenosine (CPA), 8-cyclopentyl-*N*^3^-[3-(4-(fluorosulfonyl)benzoyloxy)propyl]-*N*^1^-propylxanthine (FSCPX) and *S*-(2-hydroxy-5-nitrobenzyl)-6-thioinosine (NBTI), purchased from Sigma (St. Louis, MO, USA).

Adenosine was dissolved in 36 °C modified Krebs-Henseleit buffer (Krebs solution) containing (in mM): NaCl: 118, KCl: 4.7, CaCl_2_: 2.5, NaH_2_PO_4_: 1, MgCl_2_: 1.2, NaHCO_3_: 24.9, glucose: 11.5, ascorbic acid: 0.1, dissolved in redistilled water. CPA was dissolved in ethanol:water (1:4) solution (*v*/*v*). Dimethyl-sulfoxide (DMSO) was used as a solvent for FSCPX and NBTI. All stock solutions were adjusted to a concentration of 10 mM, except for the adenosine stock solution used to achieve 3 mM concentration in the bathing medium (it was 20 mM and was prepared always freshly before each use). Adenosine and CPA stock solutions were diluted with Krebs solution.

### 4.2. Animals, Preparations and Protocols

All experiments were carried out between 6–29 March 2018. The animal use protocols were approved by the Committee of Animal Research, University of Debrecen, Hungary (25/2013/DE MÁB). Male Hartley guinea pigs, weighing 600–800 g, were guillotined and then left atria were quickly removed and mounted at 10 mN resting tension in 10 mL vertical organ chambers (Experimetria TSZ-04; Experimetria Kft, Budapest, Hungary) containing Krebs solution, oxygenated with 95% O_2_ and 5% CO_2_ (36 °C; pH = 7.4). Atria were paced by platinum electrodes (3 Hz, 1 ms, twice the threshold voltage) by means of a programmable stimulator (Experimetria ST-02; Experimetria Kft, Budapest, Hungary) and power amplifier (Experimetria PST-02; Experimetria Kft, Budapest, Hungary). The contractile force was characterized by the amplitude of the isometric twitches, which were measured by a transducer (Experimetria SD-01; Experimetria Kft, Budapest, Hungary) and strain gauge (Experimetria SG-01D; Experimetria Kft, Budapest, Hungary) and recorded by a polygraph (Medicor R-61 6CH Recorder; Medicor Művek, Budapest, Hungary).

The atria were divided into seven groups (P1a, P1b, P2a, P2b, P2c, P3 and P4; *n* = 6–10) according to the seven experimental protocols of the present study. The protocols were the same as those used for our previous investigations [18,19], except for a rearrangement of protocol numbering and introduction of a new protocol, P2c that was carried out first in the present study (Table 1).

### 4.3. Empirical Characterization of the E/c Curves

All *E*/*c* curves were fitted to the Hill equation that is a simple and reliable empirical model of receptor function [31]:

(1) $$E=E_{\max}\times \frac{c^{n}}{c^{n}+EC_{50}{}^{n}}$$

where: *E*: The effect (that was defined as a percentage decrease in the initial contractile force of atria); *c*: The concentration of the agonist (administered for the given *E*/*c* curve); *E*_max_: The maximal effect; *EC*_50_: The agonist concentration producing half-maximal effect; *n*: The Hill coefficient (slope factor).

The Hill equation was fitted to both individual and averaged *E*/*c* curve data.

### 4.4. Quantification of the Distortion Produced by NBTI in the CPA E/c Curves

NBTI, via selective inhibition of the inward adenosine transport, increases the interstitial concentration of endogenous adenosine in the atrium under well-oxygenated conditions [26,27]. Thus, our *E*/*c* curves constructed in the presence of NBTI were distorted by a surplus interstitial adenosine concentration that was developed already before the construction of the *E*/*c* curve. To quantify this extra adenosine concentration, RRM was performed. Specifically, the averaged CPA *E*/*c* curves of P1b (only NBTI-treated) and P2c (FSCPX-NBTI co-treated) groups were fitted to Equation (2), which contained the empirical parameters of the averaged CPA *E*/*c* curve of the P1a (control) or P2b (solely FSCPX-pre-treated) group, respectively:

(2) $$E^{\prime }=100-\frac{100\times \left(100-E_{\max}\times \frac{{\left(c_{x}+c\right)}^{n}}{{\left(c_{x}+c\right)}^{n}+EC_{50}{}^{n}}\right)}{100-E_{\max}\times \frac{c_{x}{}^{n}}{c_{x}{}^{n}+EC_{50}{}^{n}}}$$

where: *E*’: The distorted effect (calculated conventionally); *E*_max_, *EC*_50_, *n*: empirical CPA *E*/*c* curve parameters (provided by Equation (1)) that define the basic (i.e., NBTI-free) conditions; *c*: The concentration of CPA (administered for the *E*/*c* curve); *c_x_*: The variable parameter of Equation (2) that indicates the CPA concentration that is equieffective with the surplus interstitial adenosine concentration accumulated by NBTI.

Determination of *c_x_* belonging to the P1b group (that characterizes the effect of NBTI treatment on the interstitial concentration of endogenous adenosine) was a part of both the original and improved forms of our receptor reserve-estimating method, while assessment of *c_x_* related to the P2c group (characterizing the effect of FSCPX-NBTI co-treatment on the interstitial level of endogenous adenosine) appeared first in the improved version.

### 4.5. Correction of Effect Values of Adenosine E/c Curves Distorted by NBTI (Improved Method)

The effect values of adenosine *E*/*c* curves biased by NBTI were corrected by means of *c_x_* values obtained from CPA *E*/*c* curves distorted by NBTI. First, the effect belonging to *c_x_* was determined using the Hill equation:

(3) $$E_{x}=E_{\max}\times \frac{c_{x}{}^{n}}{c_{x}{}^{n}+EC_{50}{}^{n}}$$

where: *E_x_*: The effect evoked solely by the extra interstitial adenosine accumulated by NBTI; *c_x_*: The appropriate theoretical CPA concentration provided by Equation (2); *E*_max_, *EC*_50_, *n*: Empirical parameters of the appropriate CPA *E*/*c* curve (provided by Equation (1)).

When *E_x_* was calculated for the correction of the averaged, only NBTI-treated adenosine *E*/*c* curve of the P4 group, *c_x_* belonging to the averaged CPA *E*/*c* curve of the P1b group (that was also solely NBTI-treated) and empirical parameters of the averaged CPA *E*/*c* curve of the P1a group (control) were substituted into Equation (3). In turn, when *E_x_* was computed for correcting the averaged, FSCPX-NBTI co-treated adenosine *E*/*c* curve of the P4 group, *c_x_* related to the averaged CPA *E*/*c* curve of the P2c group (that was FSCPX-NBTI co-treated as well) and empirical parameters of the averaged CPA *E*/*c* curve of the P2b group (that was only FSCPX-pre-treated) were written into Equation (3).

From the distorted effects and their corresponding *E_x_* values, corrected effects were computed by means of Equation (4):

(4) $$E=100\;-\frac{\left(100-E^{\prime }\right)\cdot \left(100-E_{x}\right)}{100},$$

where: *E*: The corrected effect; *E*’: The effect distorted by the surplus interstitial adenosine produced by NBTI; *E_x_*: The effect of the extra interstitial adenosine accumulated by NBTI (yielded by Equation (3)).

These corrected effect values (obtained from the procedure described above) are unique in that they reflect the action of NBTI on the adenosine *E*/*c* curve without the distortion caused by the interstitially accumulated endogenous adenosine.

### 4.6. Correction of Effect Values of Adenosine E/c Curves Distorted by NBTI (Original Method)

For the sake of comparison, the averaged, FSCPX-NBTI co-treated adenosine *E*/*c* curve of the P4 group was corrected with *c_x_* belonging to the averaged CPA *E*/*c* curve of the P1b group (that was only NBTI-treated) as well, consistent with the procedure used in our previous studies [18,19]. (The averaged, NBTI-treated adenosine *E*/*c* curve of the P4 group was corrected once, because both methods specified the same *c_x_* for this correction)

### 4.7. Data Analysis

Each atrium was required to meet three criteria in order to qualify for inclusion in the further evaluation: (i) the resting contractile force had to reach 1 mN before the first *E*/*c* curve; (ii) the mechanical activity of the paced atrium had to be regular; (iii) the response to 10 μM adenosine obtained from the first *E*/*c* curve was required to be within the mean ± 2 SD range (i.e., outliers were excluded). The mean and SD were computed using atria meeting the first two criteria. All experimental outcomes conforming to these three criteria were further processed.

According to the recommendation [32], concentrations (*c* as agonist concentration, *EC*_50_ and *c_x_*) in the equations used for curve fitting were expressed as common logarithms.

Normality of data was checked using Shapiro-Wilk normality test. Two data sets, if passed the normality test, were compared with unpaired t test. If not, Mann-Whitney U test was used. More than two data sets were compared using one-way ANOVA (with Geisser-Greenhouse correction) followed by Tukey post-testing (herein, all data sets undergone multiple comparison passed the normality test). Difference of means (or medians) was considered significant at *p* < 0.05.

Curve fitting and statistical analysis were performed with GraphPad Prism 7.04 for Windows (GraphPad Software Inc., La Jolla, CA, USA), while other calculations were made by means of Microsoft Excel 2016 (Microsoft Co., Redmond, WA, USA).

## 5. Conclusions

During the present investigation, we have refined our previous hypothesis on how FSCPX may modify the effect of NBTI on the *E*/*c* curves of adenosine receptor agonists. Based on current results, FSCPX, in addition to the permanent inactivation of the A_1_ receptor, appears to permanently inhibit an enzyme contributing to the formation of interstitial adenosine. Through this mechanism, FSCPX moderates the effects of NBTI that are mediated by increasing the interstitial concentration of endogenous adenosine before the construction of the given *E*/*c* curve.

Furthermore, we have confirmed our previous assumption that our method to assess receptor reserve for agonists with short half-life such as adenosine, in its original form, overestimates and thus overcorrects the distortion caused by NBTI, if previous FSCPX pre-treatment has occurred. Fortunately, receptor reserve estimates provided by the original method are only slightly distorted (manifested in a small overestimation of the receptor reserve). Nevertheless, use of the improved form of this method, presented herein, is recommended in the future.

## Figures and Tables

**Table 1:** The seven protocols forming seven groups /and the role of the concentration-response (*E*/*c*) curves/.

	First Incubation	First *E*/*c* Curve	Second Incubation	Second *E*/*c* Curve	Third Incubation	Third *E*/*c* Curve
P1a	Krebs solution for 25 min; 100 μM adenosine for 2 min; Krebs solution for 20 min	adenosine (1 nM–3 mM)/for comparison with the first adenosine *E*/*c* curves of groups P3 and P4/	Krebs solution for 20 min; 10 µL DMSO for 15 min	CPA (0.1 nM–0.1 mM) /NBTI-treated and its control/		
P1b			Krebs solution for 20 min; 10 µM NBTI for 15 min			
P2a			Krebs solution for 20 min; 10 µL DMSO for 45 min; Krebs solution for 60 min; 10 µL DMSO for 15 min	CPA (0.1 nM–0.1 mM) /FSCPX-pre-treated, FSCPX-NBTI co-treated and their control/		
P2b			Krebs solution for 20 min; 10 µM FSCPX for 45 min; Krebs solution for 60 min; 10 µL DMSO for 15 min			
P2c			Krebs solution for 20 min; 10 µM FSCPX for 45 min; Krebs solution for 60 min; 10 µM NBTI for 15 min			
P3		adenosine (1 nM–3 mM)/control/	Krebs solution for 20 min; 10 µM FSCPX for 45 min; Krebs solution for 75 min	adenosine (1 nM–3 mM) /FSCPX-pre-treated/		
P4			Krebs solution for 20 min; 10 µM NBTI for 15 min	adenosine (1 nM–3 mM) /NBTI-treated/	Krebs solution for 20 min; 10 µM FSCPX for 45 min; Krebs solution for 60 min; 10 µM NBTI for 15 min	adenosine (1 nM–3 mM) /FSCPX-NBTI co-treated/

The first, second and third incubation periods with the subsequent *E*/*c* curves, respectively. P1a, P1b, P2a, P2b, P2c, P3 and P4: protocol (and group) identifiers; CPA: *N*^6^-cyclopentyladenosine; NBTI: *S*-(2-hydroxy-5-nitrobenzyl)-6-thioinosine; FSCPX: 8-cyclopentyl-*N*^3^-[3-(4-(fluorosulfonyl)benzoyloxy)propyl]-*N*^1^-propylxanthine.

**Table 2:** The Hill parameters of concentration-response (*E*/*c*) curves generated with adenosine and CPA in the different groups under different conditions.

CPA	P1a (Co)	P2a (Co)	P2b (X)	P1b (NB)	P2c (X+NB)
*E*_max_	88.42 ± 0.84	89.21 ± 1.46	87.49 ± 1.75	71.68 ± 5.24 ###; ≠≠≠	84.6 ± 1.64 **
log*EC*_50_	−7.51 ± 0.09	−7.47 ± 0.074	−6.85 ± 0.05 ++	−6.53 ± 0.32 ###	−6.86 ± 0.06 §§
*n*	0.9 ± 0.06	0.98 ± 0.03	0.93 ± 0.04	0.84 ± 0.09	0.86 ± 0.02
Ado	**P3 Co**	**P4 Co**	**P3 X**	**P4 NB**	**P4 X + NB**
*E*_max_	93 ± 0.85	91.04 ± 1	93.85 ± 1.78	71.23 ± 4.9 ####; ≠≠≠≠	90.22 ± 1 ****
log*EC*_50_	−4.58 ± 0.08	−4.74 ± 0.13	−3.88 ± 0.07 +++	−5.92 ± 0.25 ####; ≠≠≠≠	−6.08 ± 0.07 §§§§; ◊◊◊◊
*n*	0.73 ± 0.03	0.85 ± 0.03	0.83 ± 0.07	0.81 ± 0.13	0.83 ± 0.1

*E*_max_, log*EC*_50_ and *n* (mean ± SEM) are best-fit values of the Hill equation (Equation (1)) fitted to the individual *E*/*c* curves. The following comparisons were made: P1a vs. P2a (CPA *E*/*c* curves); P3 vs. P4 (the first adenosine *E*/*c* curves); within the pooled P1 + P2 group (all CPA *E*/*c* curves); and within the pooled P3 + P4 group (all adenosine *E*/*c* curves in it). All significant differences are indicated (+: Co vs. X; #: Co vs. NB; §: Co vs. X + NB; ≠: X vs. NB; ◊: X vs. X + NB; *: NB vs. X + NB). Co: control (naïve); X: FSCPX pre-treatment; NB: NBTI treatment; X + NB: FSCPX-NBTI co-treatment; P1a, P1b, P2a, P2b, P2c, P3 and P4: group identifiers; Ado: adenosine; CPA: *N*^6^-cyclopentyladenosine; FSCPX: 8-cyclopentyl-*N*^3^-[3-(4-(fluorosulfonyl)benzoyloxy)propyl]-*N*^1^-propylxanthine; NBTI: *S*-(2-hydroxy-5-nitrobenzyl)-6-thioinosine; the number of marks refers to the level of statistical significance: one mark: *p* < 0.05; two marks: *p* < 0.01; three marks: *p* < 0.001; four marks: *p* < 0.0001.

**Table 3:** Influence of FSCPX and NBTI on the main empirical parameters of CPA and adenosine concentration-response (*E*/*c*) curves describing the adenosinergic control of atrial contractility.

		FSCPX	NBTI	FSCPX + NBTI
1	Effect on endogenous Ado level	*interstitial Ado level* ↓ (*by interstitial Ado production* ↓): insignificant *E*_max_ ↑ * insignificant *EC*_50_ ↓ *	**interstitial Ado level** ↑ (by inward Ado transport ↓): moderate to strong *E*_max_ ↓ moderate to strong *EC*_50_ ↑	***slight interstitial Ado level*** ↑ (*by interstitial Ado production* ↓ and inward Ado transport ↓): slight *E*_max_ ↓ slight *EC*_50_ ↑
2	Effect on exogenous Ado level	‒	interstitial Ado level ↑ (by inward Ado transport ↓): slight *E*_max_ ↑ very strong *EC*_50_ ↓	interstitial Ado level ↑ (by inward Ado transport ↓): slight *E*_max_ ↑ very strong *EC*_50_ ↓
3	Other adenosinergic effect	operating A_1_ Ado receptors ↓: insignificant *E*_max_ ↓ ** moderate *EC*_50_ ↑	‒	operating A_1_ Ado receptors ↓: insignificant *E*_max_ ↓ ** moderate *EC*_50_ ↑
1+ 3	Resulting effect on the CPA *E*/*c* curve	**moderate *EC*_50_** ↑	**moderate to strong *E*_max_** ↓ **moderate to strong *EC*_50_** ↑	**slight *E*_max_** ↓ **moderate *EC*_50_** ↑
1+ 2+ 3	Resulting effect on the Ado *E*/*c* curve	**moderate *EC*_50_** ↑	**slight to strong *E*_max_** ↓ **strong *EC*_50_** ↓	**strong *EC*_50_** ↓

The CPA and adenosine *E*/*c* curves were characterized with their two main Hill parameters (*E*_max_ and *EC*_50_), alterations of which were compared to the naïve state. Nota bene: Increase in (interstitial level of) endogenous adenosine exerts the opposite effect on the parameters of an adenosine *E*/*c* curve as an increase in (interstitial level of) exogenous adenosine (in our experimental setup). Numbers in the first column of the table: Different kinds of effects (and their combinations); **bold**: results of the present and previous studies of our team; ***bold and italic***: new results of the present study; *italic*: new suppositions made in the present study; simple formatting: established results of others and moderate conclusions drawn from results of others and our team; ↑: increase; ↓: decrease; Ado: adenosine; CPA: *N*^6^-cyclopentyladenosine; NBTI: *S*-(2-hydroxy-5-nitrobenzyl)-6-thioinosine; FSCPX: 8-cyclopentyl-*N*^3^-[3-(4-(fluorosulfonyl)benzoyloxy)propyl]-*N*^1^-propylxanthine. *: The magnitude of this effect depends on how high the level of interstitial endogenous adenosine is (herein, the resting interstitial level of endogenous adenosine is too low to elicit significant inotropic effect in the guinea pig atrium [29], thus its diminution has insignificant consequences). **: Because of the great A_1_ adenosine receptor reserve for the direct negative inotropy evoked by full agonists in the guinea pig atrial myocardium [17,18].

**Table 4:** CPA concentrations equieffective with the surplus interstitial adenosine accumulated in solely NBTI-treated eu- and hyperthyroid guinea pig atria, determined with RRM.

Ø	+T_4_	Ref.
20.4 nM	-	[26]
18.5 nM	37 nM	[27]
45.08 nM	-	[18]
38.19 nM	58.75 nM	[19]
100.2 nM	-	current study

Ø: Measurement on euthyroid atria; +T_4_: Measurement on hyperthyroid atria; CPA: *N*^6^-cyclopentyladenosine; NBTI: *S*-(2-hydroxy-5-nitrobenzyl)-6-thioinosine; RRM: Receptorial responsiveness method.

## References

[1] Dhalla A.K., Shryock J.C., Shreeniwas R., Belardinelli L. (2003). Pharmacology and therapeutic applications of A_1_ adenosine receptor ligands. Curr. Top. Med. Chem..

[2] Kenakin T.P. (2014). Techniques for More Effective and Strategic Drug Discovery. A Pharmacology Primer.

[3] Burnstock G., Fredholm B.B., North R.A., Verkhratsky A. (2010). The birth and postnatal development of purinergic signalling. Acta Physiol. (Oxf.).

[4] Burnstock G., Pelleg A. (2015). Cardiac purinergic signalling in health and disease. Purinergic Signal..

[5] IJzerman A.P., Fredholm B.B., Jacobson K.A., Linden J., Müller C.E., Frenguelli B.G., Schwabe U., Stiles G.L., Hills R., Klotz K.N. Adenosine receptors. IUPHAR/BPS Guide to Pharmacology.

[6] Shryock J.C., Belardinelli L. (1997). Adenosine and adenosine receptors in the cardiovascular system: Biochemistry, physiology and pharmacology. Am. J. Cardiol..

[7] Szentmiklosi A.J., Cseppento A., Harmati G., Nanasi P.P. (2011). Novel trends in the treatment of cardiovascular disorders: Site- and event- selective adenosinergic drugs. Curr. Med. Chem..

[8] Headrick J.P., Ashton K.J., Rose′meyer R.B., Peart J.N. (2013). Cardiovascular adenosine receptors: Expression, actions and interactions. Pharmacol. Ther..

[9] Lasley R.D. (2018). Adenosine Receptor-Mediated Cardioprotection-Current Limitations and Future Directions. Front. Pharmacol..

[10] Scammells P.J., Baker S.P., Belardinelli L., Olsson R.A. (1994). Substituted 1,3-dipropylxanthines as irreversible antagonists of A_1_ adenosine receptors. J. Med. Chem..

[11] Srinivas M., Shryock J.C., Scammells P.J., Ruble J., Baker S.P., Belardinelli L. (1996). A novel irreversible antagonist of the A_1_-adenosine receptor. Mol. Pharmacol..

[12] Srinivas M., Shryock J.C., Dennis D.M., Baker S.P., Belardinelli L. (1997). Differential A_1_ adenosine receptor reserve for two actions of adenosine on guinea pig atrial myocytes. Mol. Pharmacol..

[13] Morey T.E., Belardinelli L., Dennis D.M. (1998). Validation of Furchgott′s method to determine agonist-dependent A_1_-adenosine receptor reserve in guinea-pig atrium. Br. J. Pharmacol..

[14] Baker S.P., Scammells P.J., Belardinelli L. (2000). Differential A(1)-adenosine receptor reserve for inhibition of cyclic AMP accumulation and G-protein activation in DDT(1) MF-2 cells. Br. J. Pharmacol..

[15] Lorenzen A., Beukers M.W., van der Graaf P.H., Lang H., van Muijlwijk-Koezen J., de Groote M., Menge W., Schwabe U., IJzerman A.P. (2002). Modulation of agonist responses at the A(1) adenosine receptor by an irreversible antagonist, receptor-G protein uncoupling and by the G protein activation state. Biochem. Pharmacol..

[16] Bozarov A., Wang Y.Z., Yu J.G., Wunderlich J., Hassanain H.H., Alhaj M., Cooke H.J., Grants I., Ren T., Christofi F.L. (2009). Activation of adenosine low-affinity A_3_ receptors inhibits the enteric short interplexus neural circuit triggered by histamine. Am. J. Physiol. Gastrointest. Liver Physiol..

[17] Gesztelyi R., Kiss Z., Wachal Z., Juhasz B., Bombicz M., Csepanyi E., Pak K., Zsuga J., Papp C., Galajda Z. (2013). The surmountable effect of FSCPX, an irreversible A(1) adenosine receptor antagonist, on the negative inotropic action of A(1) adenosine receptor full agonists in isolated guinea pig left atria. Arch. Pharm. Res..

[18] Kiss Z., Pak K., Zsuga J., Juhasz B., Varga B., Szentmiklosi A.J., Haines D.D., Tosaki A., Gesztelyi R. (2013). The guinea pig atrial A_1_ adenosine receptor reserve for the direct negative inotropic effect of adenosine. Gen. Physiol. Biophys..

[19] Pak K., Papp C., Galajda Z., Szerafin T., Varga B., Juhasz B., Haines D., Szentmiklosi A.J., Tosaki A., Gesztelyi R. (2014). Approximation of A_1_ adenosine receptor reserve appertaining to the direct negative inotropic effect of adenosine in hyperthyroid guinea pig left atria. Gen. Physiol. Biophys..

[20] Thorn J.A., Jarvis S.M. (1996). Adenosine transporters. Gen. Pharmacol..

[21] Deussen A., Stappert M., Schäfer S., Kelm M. (1999). Quantification of extracellular and intracellular adenosine production: Understanding the transmembranous concentration gradient. Circulation.

[22] Deussen A., Weichsel J., Pexa A. (2006). Features of adenosine metabolism of mouse heart. Purinergic Signal..

[23] Gesztelyi R., Zsuga J., Juhász B., Dér P., Vecsernyés M., Szentmiklósi A.J. (2004). Concentration estimation via curve fitting: Quantification of negative inotropic agents by using a simple mathematical method in guinea pig atria. Bull. Math. Biol..

[24] Grenczer M., Pinter A., Zsuga J., Kemeny-Beke A., Juhasz B., Szodoray P., Tosaki A., Gesztelyi R. (2010). The influence of affinity, efficacy and slope factor on the estimates obtained by the receptorial responsiveness method (RRM): A computer simulation study. Can. J. Physiol. Pharmacol..

[25] Zsuga J., Erdei T., Szabó K., Lampe N., Papp C., Pinter A., Szentmiklosi A.J., Juhasz B., Szilvássy Z., Gesztelyi R. (2017). Methodical Challenges and a Possible Resolution in the Assessment of Receptor Reserve for Adenosine, an Agonist with Short Half-Life. Molecules.

[26] Karsai D., Zsuga J., Juhász B., Dér P., Szentmiklósi A.J., Tósaki A., Gesztelyi R. (2006). Effect of nucleoside transport blockade on the interstitial adenosine level characterized by a novel method in guinea pig atria. J. Cardiovasc. Pharmacol..

[27] Karsai D., Gesztelyi R., Zsuga J., Jakab A., Szendrei L., Juhasz B., Bak I., Szabo G., Lekli I., Vecsernyes M. (2007). Influence of hyperthyroidism on the effect of adenosine transport blockade assessed by a novel method in guinea pig atria. Cell Biochem. Biophys..

[28] Gesztelyi R., Zsuga J., Cseppento A., Bajza A., Varga A., Szabó J.Z., Szentmiklósi A.J. (2003). Special sensitization pattern in adenosine-induced myocardial responses after thyroxine-treatment. J. Pharmacol. Sci..

[29] Pak K., Zsuga J., Kepes Z., Erdei T., Varga B., Juhasz B., Szentmiklosi A.J., Gesztelyi R. (2015). The effect of adenosine deaminase inhibition on the A_1_ adenosinergic and M_2_ muscarinergic control of contractility in eu- and hyperthyroid guinea pig atria. Naunyn Schmiedebergs Arch. Pharmacol..

[30] Pavan B., IJzerman A.P. (1998). Processing of adenosine receptor agonists in rat and human whole blood. Biochem. Pharmacol..

[31] Gesztelyi R., Zsuga J., Kemeny-Beke A., Varga B., Juhasz B., Tosaki A. (2012). The Hill equation and the origin of quantitative pharmacology. Arch. Hist. Exact Sci..

[32] GraphPad Software Inc. (2016). GraphPad Curve Fitting Guide.

